# Analysis of the Blood in a Case of Lead Colic

**Published:** 1845-01

**Authors:** 


*Analysis of the Blood in a case of Lead Colic.* By Professor Cozzi.—Professor Cozzi, in analysing the blood of a person severely affected with Lead Colic, discovered that the lead existed in the state of a salt or of an oxide of the metal. He at the same time ascertained that the lead was neither united with the hæmatosin nor fibrin, but was wholly contained in the albumen of the blood.—*Ed. Med. Surg. Journ., from Journ. de Pharmacie.*

